# Elimination of lymphatic filariasis as a public health problem from Tonga

**DOI:** 10.1186/s41182-019-0169-2

**Published:** 2019-07-15

**Authors:** Reynold `Ofanoa, Tukia Ofa, E. A. Padmasiri, D. Ramaiah Kapa

**Affiliations:** 1 Public Health Division, Ministry of Health, Nuku’alofa, Tonga; 2 Formerly with Division for Pacific Technical Support, World Health Organization, Suva, Fiji; 3 Formerly with Vector Control Research Centre (ICMR), Indira Nagar, Pondicherry, 605006 India

**Keywords:** Lymphatic filariasis, *Wuchereria bancrofti*, Elimination, PacELF, Tonga

## Abstract

**Background:**

Tonga was highly endemic for lymphatic filariasis (LF) caused by diurnally sub-periodic *Wuchereria bancroft*i transmitted by *Aedes* vector species. LF prevalence declined very appreciably as a result of chemotherapeutic intervention measures implemented in 1977, but low levels of infection persisted. Along with other Pacific Island countries and in partnership with the Pacific Programme to Eliminate LF (PacELF), Tonga implemented a programme to eliminate LF as a public health problem.

**Methods:**

On the basis of historical data and baseline survey, all the divisions of the country were declared as endemic. Five to six consecutive rounds of effective MDA were implemented in all the divisions during 2001–2006. The impact of MDA was assessed through interim and post-MDA antigen (Ag) detection surveys among adults and transmission assessment surveys among children. The chronic disease burden was assessed by health workers through observation.

**Results:**

The base-line Ag prevalence was 2.70%. The treatment coverage was > 80% in all MDA rounds. The mid-term surveys showed an Ag prevalence of 2.46%. The pre-stop MDA Ag survey revealed an Ag prevalence of 0.34%. The stop MDA survey and transmission assessment surveys among children showed Ag prevalence at < 0.05%, indicating transmission is negligible. Health workers concluded that filarial lymphedema or hydrocele condition in the communities is absent or very rare.

**Conclusion:**

Tonga had successfully met the criteria for elimination of LF as a public health problem. The accomplishment was acknowledged by the WHO in 2017. Tonga looks forward to work with stakeholders to eliminate transmission of LF and achieve zero incidence of infection.

## Introduction

Lymphatic filariasis (LF) is a significant public health problem in different regions of the world. The disease is widely prevalent in the Western Pacific region and South Pacific regions. Within the South Pacific region, 16 countries including the Kingdom of Tonga are endemic for LF. The epidemiology of LF in the region is characterized by the prevalence of both nocturnally periodic and diurnally sub-periodic races of *W. bancrofti* and involvement of *Aedes*, *Anopheles*, and *Culex* vectors [1]. The most notable feature is a very high prevalence of *Aedes*-transmitted diurnally sub-periodic *W. bancrofti* in several countries including Tonga [1]. Tonga implemented intervention measures in the year 1976, leading to a dramatic decline of microfilaria (Mf) rate. However, residual infection persisted and remained as a challenge [2].

In the year 2000, the WHO launched a Global Programme to Eliminate LF and it envisaged elimination of LF as a public health problem [3]. The goals of the programme are (i) interruption of transmission in all endemic communities using mass drug administration (MDA) and (ii) alleviation of suffering among people affected with chronic disease using morbidity management and disability prevention (MMDP) measures. WHO and various other stakeholders encouraged and supported the endemic countries to implement the MDA and MMDP interventions and eliminate LF. This significant development received further impetus in the South Pacific region by a regional programme initiative called the Pacific Programme to Eliminate LF (PacELF) [4]. This umbrella organization of 22 countries of the region provided logistics and technical support and guided launching and implementation of the national programmes to eliminate LF.

Tonga is an active proponent of PacELF and launched the national programme to eliminate LF in the year 2000. The implementation of the programme and its outcomes are presented in this paper.

### Geography and population

**Fig. 1:**
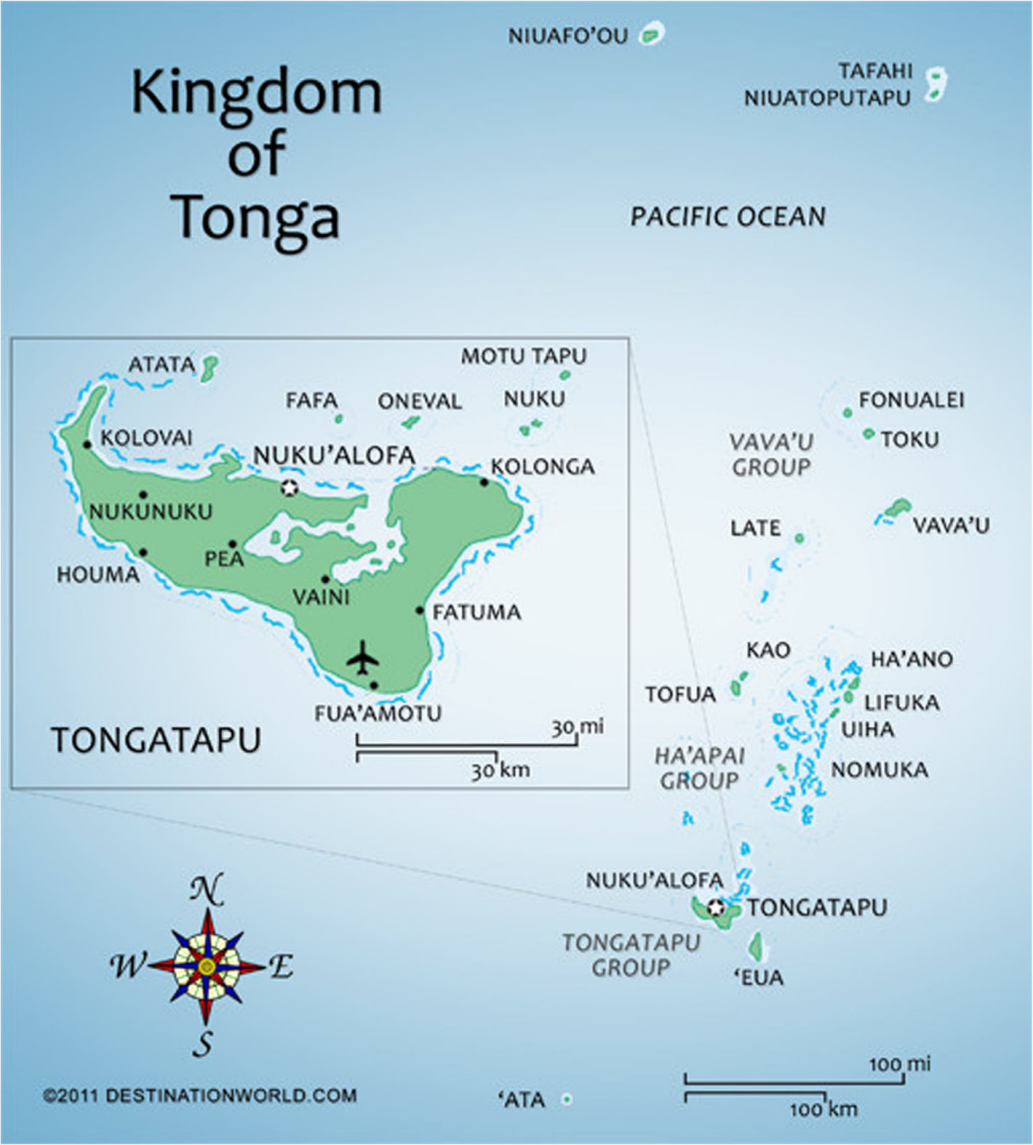
Map of Tonga

The Kingdom of Tonga is an archipelago in the South Pacific Ocean. Covered with tropical rainforest, Tonga consists of 176 islands, geographically divided into three groups—Tongatapu in the south, Ha’apai in the centre, and Vava’u in the north. Isolated islands include Niuafo’ou, Niuatoputapu, and Tafahi (together known as the Niuatoputapu or Niuas island group) in the far north and ʿAta in the far south (Fig. 1). Of the 176 islands, 36 are inhabited and Tongatapu is the largest and most populated island and includes the capital city of Nuku’alofa. For administration convenience, the country is divided into five divisions (Table 1) and there were a total of 22 districts. The climate of Tonga is tropical throughout the year. The total rainfall is higher in the most northern islands (2500 mm) and less in southern islands (1700 mm). Mean annual temperature ranges from 23 to 28^°^C and mean humidity persists around 75%.

**Table 1:** Enumerated population and area of different divisions of Tonga

Division (island group)	Population (2011)	Population density (2011)	Area (km^2^)
Tongatapu	75,416	274	275.5
Vava’u	14,922	93	161.0
Ha’apai	6616	50	132.1
‘Eua	5016	57	88.3
Ongo Niua	1282	18	72.0

Source: Tonga 2011 census of population and housing

About 98% of Tongans are Polynesian with a very small mixture of Melanesian people. As of 2011, the population of Tonga was 103,252. The division of Tongatapu accounts for 73% of the population and others 27% (Table 1). Tonga achieved 98.9% literacy rate. Tonga has a small, open, and South Pacific island economy. The economy is characterized by subsistence agriculture, vulnerability to natural hazards, and significant reliance on external income that includes donor aid and remittances. The standard of living has improved considerably over the last 50 years, and there is now little absolute poverty. The country is placed 95 in the United Nations Development Programme’s Human Development Index (HDI), one of the highest ranking of any Pacific island state. This high ranking reflects the comparatively high GNI per capita of US$4260 (2014), the high life expectancy, and the near-universal literacy.

### Health care delivery

The Ministry of Health (MOH) is responsible for the delivery of preventative and curative health services in the country. MOH’s mission is to support and improve the health of the nation by providing quality, effective, and sustainable health services and being accountable for health outcomes. Tonga’s population has very good access to health care and high standard of health. Tonga has made good progress towards achieving the health-related Millennium Development Goal indicators for maternal and child mortality. This reflects Tonga’s effective primary healthcare delivery and public health infrastructure. Health care is delivered in Tonga’s island divisions through (i) one main referral hospital, (ii) three community hospitals, (iii) 14 health centres, and (iv) 34 reproductive and child health clinics. The health care network covers well the entire population [5].

### Prior LF situation

LF in Tonga is caused by diurnally sub-periodic *Wuchereria bancrofti* and is transmitted by two species of mosquitoes, *Aedes tongae* [6] and *Aedes tabu* [7–9]. The prevalence of LF was very high in different islands of Tonga for centuries (Table 2). A comprehensive study conducted in 1976 by Desowitz et al. [2] in Te’ekiu village, Tongatapu island, and Pangai Island, Ha’apai group, provides an insight into the magnitude of LF problem. In this study, the Mf prevalence was found to be 45% and the geometric mean density of Mf ranged from 1.00 to 56.80 in different age groups. Of the examined people in different age groups, 50 to 94% showed skin test positivity with Sawada’s FST-31 *Dirofilaria immitis* antigen (Ag). The hydrocele prevalence was found to be as high as 55% and lymphedema/elephantiasis (locally known as Kulakula) prevalence 8.0%.

**Table 2:** The Mf rate and disease rate reported from various studies in Tonga

Place	Year	No of people tested for Mf	Mf rate (%)	Author
Nomuka	1896	Data not available	28.8	Thorpe, 1896, in [1]
Lifuka	1896	Data not available	46.9	Thorpe, 1896, in [1]
Vava’u	1896	Data not available	20.0	Thorpe, 1896, in [1]
Tongatapu	1896	Data not available	29.2	Thorpe, 1896, in [1]
Tongatapu	1925	Data not available	13.5	Hopkins, 1925, in [6]
Ha’apai	1925	Data not available	14.3	Hopkins, 1925, in [6]
Vava’u	1925	Data not available	46.2	Hopkins, 1925, in [6]
Tongatapu	1957	Data not available	28.2–48.5	Tapa, 1957, in [10]
Vava’u	1957	Data not available	49.6	Tapa, 1957, in [10]
Hihifo village in Niuatoputapu	1970	680 (all age groups)	16.4 (blood smear)	[11]
Hihifo village in Niuatoputapu	1970	55 (children of 5–9 years)	71.0 (membrane filtration)	[11]
Te’ekiu village, Tongatapu island	1976	297	45.0 (combined for both the areas)	Desowitz et al. 1976 [2]
Pangai Island, Ha’apai group		309		
Entire country	1976	9882	17.4	Country report cited in The PacELF Way, WHO, 2006 [4]
Entire country^a^	1979	9676	1.0	Country report cited in The PacELF Way, WHO, 2006 [4]
Some areas of the country	1983–1984	4875	0.4	Country report cited in The PacELF Way, WHO, 2006 [4]
Tongatapu (Vaiola Hospital and Lapaha, Vaini, Fua’amotu and Houma health centres)	1998–1999	1584	0.6	MOH, 1999, unpublished data [12]

^a^Mass treatment implemented in the entire country in 1977

A country-wide Mf prevalence survey, using thick blood smear technique, in 1976, showed an Mf prevalence of 17.4% (*n* = 9882). In an effort to control LF, a mass treatment programme was initiated in May 1977. Under the programme, a single dose of DEC was given once a month and in total 12 doses were given over a period of 12 months [4]. A post-treatment nation-wide survey in 1979 showed an Mf rate of 1.0% (*n* = 9676), indicating an appreciable impact of the mass treatment. A follow-up Mf survey carried out during 1983–1984 showed an Mf rate of only 0.35% (17/4875), suggesting a further decline from the level observed in 1979. Further surveys carried out in 1998–1999 revealed an Mf rate of 0.63% (10/1584), suggesting persistence of residual infection (Table 2).

### LF elimination programme

Following the 1997 World Health Assembly Resolution on LF elimination and launching of the Pacific Programme to eliminate lymphatic filariasis (PacELF) [4], the MOH, Tonga, decided to launch a national LF elimination programme.

The programme was coordinated by the Director of Health and managed by the Chief Medical Officer, Public Health. The medical officers of the three health districts—Vava’u, Ha’apai, and ‘Eua—extended support in implementing the programme in respective districts. The programme was implemented through Reproductive and Child Health clinics and Health Centres. The objectives of the programme were (i) to achieve 100% geographic coverage with MDA in the year 2001, (ii) to implement five effective rounds of MDA throughout the country, and (iii) to achieve interruption of transmission by 2005.

To implement the NPELF, particularly the MDA, each division/island group was designated as an IU. Thus, there were five IUs—and these included ‘Eua, Ha’apai, Ongo Niua, Tongatapu, and Vava’u.

#### Delineation of endemicity

On the eve of launching the programme to eliminate LF, the MOH carefully analyzed the LF historical data and the LF situation in the country. Besides, a nation-wide Ag survey was carried out, using immunochromatographic card test (ICT) (Alere: Binax Now), in 1999–2000, to identify the endemic areas to implement the MDA programme. Subsequently, this survey was considered as a survey of monitoring and evaluation (M & E), and its outcome was used as baseline data for MDA programme (see the “M & E” section).

#### MDA implementation

Starting from 2001, five consecutive rounds of annual MDA were implemented throughout the country during 2001–2005. An additional MDA was implemented in 2006 in the Niuas group of islands. MDA was implemented as effectively as possible to meet the stiff LF elimination criteria for *Aedes* vector areas, i.e., < 1.0% Ag prevalence in 6–7-year-old children [13].

For the implementation of MDA, each of the five divisions was considered as an implementation unit (IU). As per the WHO and PacELF guidelines, diethylcarbamazine (DEC) + albendazole (ALB) combination therapy was used in the MDA programme. The quantum of drugs required for the five IUs was estimated by the MOH on the basis of population size. The drugs were procured well in advance and stored in Tongatapu. ALB was procured for each round of MDA from the donor pharmaceutical company, GlaxoSmithKline, through WHO/PacELF facilitation. DEC (in 50 mg formulation) was procured by the MOH through the Japan International Cooperation Agency as a donation. The drugs required for each island group were sent from Tongatapu at least 2–3 weeks prior to the drug distribution date.

The national programme, in consultation with different island group health personnel, developed the drug distribution guidelines and strategies. Community leaders, elders, nobles, and religious leaders and heads of community-based organizations, who play an important role in decision-making and influencing the opinion, were co-opted to support the MDA. The drugs were distributed with the help of government census data (1996). Using this data, health workers visited the households in each community and prepared a register for each household. Drugs were distributed with the help of the registers and the drug distribution and drug consumption details were recorded for each household.

In each island, the local health personnel, with the support of central team members, distributed the drugs on the main island and peripheral islands of each division. In each division, for each district, the district nurse was made in charge of drug distribution. He/she organized 5–6 teams and each team consisted of 3–4 health personnel from among the reproductive health nurses in hospitals and health centres, clinical nurses and nursing practitioners, and pharmacists. No community volunteers were involved in the drug distribution.

Drugs were delivered in central places, particularly churches, as a significant proportion of people regularly attend the church services. The church encouraged the communities to actively participate in the programme. Schools were used to distribute the drugs to children and higher-class students. Those who missed the treatment in churches and schools were informed to gather in community halls and were provided drugs. In order to further improve the treatment coverage, house to house visits were made to deliver the drugs to those people who missed treatment in central places. Throughout the programme, directly observed treatment was practiced. Drug distribution activity in each division required 3–4 weeks, as teams of only the health workers distributed the drugs and travel to smaller islands was time-consuming. At times, bad weather delayed the drug distribution activity and held up the teams in smaller islands for days together. Prior to launching the programme, two staff from MPH received training on MDA at PacELF, Suva, Fiji. The staff conducted a training programme at MPH, Nuku’alofa, to medical officers of different divisions. The medical officers imparted training to various categories of health personnel in their respective divisions.

The drugs were given at the WHO recommended dose—DEC at 6 mg/kg body weight and one tablet of ALB (400 mg). During each round of drug distribution, drugs were given according to the age of the person. The dosage of drugs for different age groups was determined on the basis of relationship between age and weight of the population. Children < 3 year age, pregnant women, seriously ill, and people aged > 80 years were excluded from MDA. The incidence of side reactions was monitored among treated communities.

The drug distribution activity was supported by information, education, and communication campaign on TV, radio, and newspapers. In churches, community notices on MDA were read out by priests and ministers to the assembled people. Pamphlets in the local language, highlighting the public health importance of LF and the objectives of MDA, were distributed. Community meetings were held to explain the purpose of the MDA programme. Most of the drug distribution activity was completed on day one of the programme. However, people who missed the treatment were visited at household and given the treatment during the following days and weeks.

Adverse events were very rare in the treated population during different rounds of MDA. There were very few cases of headache or lethargy. The symptoms were so mild that no response from the health system was required. In very few cases, treatment with paracetamol was advised.

#### M & E

Epidemiological M & E of the MDA programme had been a key component. The programme followed the M & E guidelines envisaged by the PacELF and outlined in PacELF monitoring and analysis network [4]. The M & E included Ag surveys, conducted at four time points as described below.

##### A survey

It is a baseline assessment of LF Ag prevalence in the country, using a protocol of convenience sampling of the adult population in sentinel sites. The survey was conducted in 1999–2000, which is prior to MDA.

##### B survey

It is a mid-term evaluation of Ag prevalence among the adult population in sentinel sites to assess if the MDA programme had desired effect. The survey was conducted during December 2003–August 2004, i.e., a few months after completing the third round of MDA.

##### C survey or pre-stop MDA survey

It envisages a thorough final evaluation of Ag prevalence covering all areas of the country. Its objective is to gauge if Ag prevalence fell below the threshold level of 1% in the cross-sectional survey and facilitate decision on stopping or continuing MDA. The C survey was conducted in 2006, i.e., after completion of five rounds of nationwide MDA in 2005 and sixth MDA in Nuas island in 2006. Purposefully, all the sites/villages were surveyed in the Niuas division, where a high baseline Ag prevalence was recorded. In Niuas, Ag assessment was done in all 12 sites (100%), and in each of the other four divisions, 4–6 sites were chosen.

##### D survey or stop MDA survey

Its purpose is to assess Ag prevalence in young children to gauge if transmission interruption is achieved and MDA can be stopped. After completing five rounds of MDA in the entire country and C survey in 2005–2006, the national programme implemented a nationwide D survey. The D survey is equivalent to transmission assessment survey 1 (TAS 1) in the current M & E guidelines of the WHO [13]. In accordance with the guidelines of the D survey, the survey was conducted among the children of six years. The survey was school based and conducted among children of 1st grade, most of whom are six years old. Prior to the survey, school principals were contacted through the Ministry of Education and informed the objectives of the survey. An Ag prevalence of < 1.0% among the surveyed children is considered as an indicator of transmission interruption and stopping the MDA.

Tonga implemented all the above surveys and the results are presented below. All Ag surveys were carried out using the ICT cards, which were procured and supplied by PacELF. The ICT cards were stored and tests conducted in the field per the instructions given in the manufacturer’s brochure.

#### Post-MDA surveillance surveys

The current post-MDA surveillance guidelines of the WHO recommend conducting TAS twice, i.e. TAS 2 and TAS 3. TAS 2 is to be conducted 2–3 years after TAS 1 or stop MDA survey and TAS 3 after 2–3 years of implementing TAS 2. The guidelines recommend testing of 6–7-year-old children drawn from 30 to 40 schools or communities of an evaluation unit (EU). If the number of children found positive for Ag is equivalent or less than the critical cutoff value, the prevalence rate of < 1.0% had been determined as the critical cutoff value and this level is considered to sustain transmission interruption [13].

As part of the post-MDA surveillance, TAS 2 was conducted in 2011 (it was due in 2010, but could not be conducted due to logistic reasons), i.e., four years after stopping the MDA. Epidemiologically, leaving a gap of four years between stop MDA survey and TAS 2 is good because it enables detection of new infections, if any, occurred over a longer period of four years. TAS 2 was also conducted in all the five divisions, which were together considered as one EU. It was conducted among school children, as the enrolment rate was > 75% [13]. In the schools, first grade students were blood tested to assess Ag prevalence, using ICT cards. Approximately, a total of 3100 students were enrolled in first grade in all the five divisions. To make the TAS outcome very robust, all the first grade students attending the school were tested for Ag. In TAS 2, a total of 2451 students were blood tested.

TAS 3 was conducted following the same methodology as in TAS 2. It was conducted in 2015, i.e., about 4 years after conducting TAS 2. In TAS 3, a total of 2883 students were blood tested.

#### Treatment of Ag-positive individuals

As and when Ag-positive individuals were detected in any survey, they were treated with single dose of DEC + ALB. They were advised to undergo further blood testing for Mf or Ag and take treatment if found positive.

#### Data collection and management

The district level health officers were responsible and coordinated data collection in different districts. They transferred the original data forms to the division level health officer, who transferred the data to the central programme manager. The programme manager organized the data and undertook analyses from time to time.

## Results

### A survey (baseline survey) (1999–2000)

A total of 4002 people were surveyed for Ag, and the Ag prevalence rate was found to be 2.7%, ranging from 0.0% in ‘Eua to 37.7% in Nuas. This Ag prevalence of 2.7% was higher than the threshold level (1.0%) for areas endemic for LF transmitted by *Aedes* species [13]. Three factors—(i) history of high prevalences of LF in various parts of the country (Table 2), (ii) persistence of 1.0% Mf prevalence after intervention measures in 1977, and (iii) 2.70% Ag prevalence observed in the baseline survey—prompted the MOH to follow a cautious approach and declare that LF continues to persist in the country. Therefore, the MOH declared that the entire country is endemic for LF, and an MDA-based LF elimination programme will be implemented throughout the country.

### MDA (2001–2006)

In total, five rounds of MDA were implemented throughout the country during 2001–2006. The geographic coverage of the programme was 100% from the first year of the MDA programme (Table 3). During each of the five MDAs, very effective treatment coverage was achieved. The programme drug coverage ranged from 81.6% in 2001 to 90.8% in 2003 (Table 3). There were no reports from health centres on any group of people or any village consistently refusing treatment. Thus, systematic non-compliance was never an issue for the programme.

**Table 3:** Summary of national MDA data by year for Tonga

Year	Population requiring PC for LF	Number of IUs covered	Geographical coverage (%)	Total population of IUs	Reported number of people treated	Programme (drug) coverage (%)
2001	98,000	5	100.00	98,036	79,969	81.6
2002	98,000	5	100.00	90,720	82,023	90.4
2003	98,000	5	100.00	97,784	88,752	90.8
2004	98,000	5	100.00	97,784	83,719	85.6
2005	98,000	5	100.00	98,000	83,218	84.9
2006	1002	1	100.00	1002	923	92.1

### Mid-term survey (2003–2004)

The mid-term survey was conducted in all five divisions. A total of 3294 people were assessed for Ag, and the number tested ranged from 858 to 1043 in different divisions. The overall Ag prevalence was 2.46% and the prevalence in different islands ranged from 0.0 to 6.98% (Table 4).

**Table 4:** Results of mid-term Ag survey in Tonga, 2003–2004

Division	Number of people tested	Number positive for Ag	Ag prevalence (%)
Tongatapu	533	0	0.0
Vava’u	1043	7	0.67
Ha’apai	858	14	1.63
‘Eua	Not done	–	–
Niuas	860	60	6.98
Total	3294	81	2.46

### C survey (pre-stop MDA survey, 2006)

A total of 2927 people were tested and the number tested in different divisions ranged from 451 to 630. The sample of 2927 was drawn from 31 communities in five divisions. The overall Ag prevalence was 0.34%. While Niuas showed Ag prevalence of 0.46% (5/(630 + 463)) (range 0.0–1.16%), Ha’apai showed 1.07% (range 1.87–4.00%). All the other divisions showed 0.0% (Table 5).

**Table 5:** Detailed results of C survey in Tonga, 2006

Division	Site	Population	Number of people tested	Number positive for Ag	Ag prevalence (%)
Tongatapu	Tatakamotonga	1743	146	0	0
	Veitongo	952	130	0	0
	Sia’atoutai	536	95	0	0
	Kolomotu’a	2010	120	0	0
	Total	5241	491	0	0
Vava’u	Vaimalo	84	60	0	0
	Kapa	63	42	0	0
	Toula	288	164	0	0
	Neiafutahi	251	137	0	0
	Nuapapu	177	48	0	0
	Total	863	451	0	0
Ha’apai	Fakakakai	115	107	2^a^	1.87
	Faleloa	532	111	0	0
	Holopeka	183	65	0	0
	Koulo	285	53	0	0
	Fotua	208	75	3^a^	4.00
	O’ua	159	57	0	0
	Total	1482	468	5	1.07
‘Eua	Angaha	369	107	0	0
	Mata’aho	228	99	0	0
	Fata’ulua	249	95	0	0
	Ha’atu’a	483	123	0	0
	Total	1329	424	0	0
Niua’s—Niuatoputapu	Hihifo	385	240	2^b^	0.83
	Falehau	248	185	1^b^	0.54
	Vaipoa	263	173	2	1.16
	Tafahi	95	32	0	0
	Total	991	630	5	0.79
Niua’s—Niuafo’ou	Mu’a	24	24	0	0
	Sapa’ata	138	86	0	0
	Petani	95	66	0	0
	Fata’ulua	73	52	0	0
	Esia	161	87	0	0
	Mata’aho	24	24	0	0
	Kolofo’ou	145	82	0	0
	Tongamama’o	65	42	0	0
	Total	715	463	0	0
Total		10,621	2927	10	0.34

^a^Blood examination showed negative result for Mf

^b^Blood examination showed Mf in thick smears. In Hihifo, only 1 of the 2 Ag-positive individuals showed Mf

The Ag-positive individuals were blood tested for Mf. Of the five Ag-positive individuals found in Niuatoputapu, Niuas, one each in the two communities was found positive for Mf. All the five Ag-positive individuals in Ha’apai were negative for Mf.

### D survey (TAS 1/stop MDA survey, 2007)

The survey was conducted in all five divisions. Of the 3283 children registered in schools, 2391, equivalent to 72.8%, were tested for Ag and none of the children were found positive (Table 6) The results clearly suggest that transmission is totally interrupted in every group of islands. Thus, the major objective of the programme—transmission interruption—has been achieved. Accordingly, it was decided to stop the MDA programme.

**Table 6:** Results of D survey conducted in different divisions in Tonga

Island group	Number of children registered in schools	Number of children tested	% tested	Number of children positive for Ag
Tongatapu (Central)	1202	725	60.3	0
Tongatapu (East)	554	409	73.8	0
Tongatapu (West)	478	375	78.5	0
‘Eua	189	169	89.4	0
Ha’apai	272	219	80.5	0
Vava’u	504	413	81.9	0
Niua’s	84	81	96.4	0
Total	3283	2391	72.8	0

### Post-MDA surveillance surveys (2011 and 2015)

#### TAS 2

A total of 2451 students drawn from all five divisions were tested for Ag and none of them was found positive, and the Ag prevalence was 0.0%. This result indicates that transmission interruption, evident from the results of D survey (TAS 1) that revealed 0% Ag prevalence, sustained from 2007 to 2011 period.

#### TAS 3

A total of 2806 children from all the five divisions were tested for Ag and one child was found positive, and the Ag prevalence was 0.04%. The positive child belongs to the Niua’s division. This extremely low level of Ag prevalence suggests that transmission interruption continued to be sustained over a period of 2007 to 2015.

### MMDP

After the implementation of a nation-wide mass treatment programme in 1977, the Mf rate declined dramatically and remained at about 1.0% level. The studies and surveys conducted in subsequent years showed that prevalence of Mf continued to be low. Simultaneously, there has been a tremendous decline also in the prevalence of chronic disease, both lymphoedema and hydrocele. Currently, people with lymphoedema and hydrocele condition are rare and the younger generation is completely free from the disease.

## Discussion

Tonga had a history of a high prevalence of microfilaraemia and chronic disease. The hydrocele prevalence of 55% recorded in 1976 was among the highest observed in LF endemic areas (Table 2). Effective intervention measures implemented in 1976 had a dramatic impact on Mf prevalence. The reduced levels of Mf prevalence were sustained, which is evident from the relatively low Ag prevalence (2.70%) recorded in 1999–2000, when the baseline Ag prevalence was assessed for LF elimination programme. However, this Ag prevalence is higher than the recommended threshold level of 1.0% for *Aedes* vector areas. Left untreated, this low-level Ag prevalence may continue to persist for several years. Hence, Tonga had chosen to implement the MDA programme to reduce the infection to below threshold level and eliminate LF as a public health problem.

The MOH had implemented very effective MDA, evident from very high treatment coverage rates over the six year period. Ag prevalence of 0% in stop MDA survey and TAS 2 and < 0.1% in TAS 3, over a period of eight years (2007–2015), clearly suggest that the LF transmission in Tonga has been interrupted by the MDA programme. Such impressive results from all over the country also suggest that there are no hotspots in the country and LF is unlikely to resurge. The programme has met all the LF elimination criteria for interruption of transmission viz., (a) five consecutive rounds of effective MDA and (b) reduction and sustenance of Ag prevalence in children to < 1.0% [14]. Thus, the Kingdom of Tonga achieved total interruption of transmission of LF.

A few research studies also confirmed that Tonga eliminated LF as a public health problem. To examine the application of CELISA antifilarial IgG4 antibody (Ab) assay in surveillance of the LF elimination programme, Joseph et al. [15] conducted a study in three countries including Tonga. Under the study, 797 school children aged 5 to 6 years were tested for Ag using ICT and those that tested ICT positive were re-examined for Mf. The children were drawn from schools in ‘Eua, Ha’apai, and Vava’u. Filter paper samples were also collected for Ab serology. The study showed Mf and Ag prevalence of 0% and Ab prevalence of 6.0% in Tonga. It confirmed “cessation of transmission” in Tonga. The higher Ab prevalence was attributed to sensitivity and specificity of the filariasis CELISA for filter paper sampling, which at the time gave false-positive results at a rate of about 40% [15].

In a study on the feasibility of synchronous assessment of STH with TAS, Chu et al. [16] tested 1800 children from 74 schools in Tongatapu and 634 children from 53 schools in Vava’u and Ha’apai for LF Ag prevalence, using ICT cards. The study showed an overall Ag prevalence of 0.3% (7/2434). The prevalence was 0.2% (3/1800) in Tongatapu and 0.6% (4/634) in Vava’u and Ha’apai. These prevalences were well below the critical cutoff value of < 1.0%. This study also confirmed the sustenance of interruption of transmission of LF in Tonga.

Elimination of LF in Tonga is considered as a significant success, because in some countries of the region such as French Polynesia [17] and American Samoa [18], the low levels of prevalence and transmission of LF persisted despite intensive intervention measures. This persistence infection is attributed to the inherent high capacity of the *Aedes* vectors to perpetuate transmission [19]. Nevertheless, the success in Tonga and also Cook Islands [20] demonstrates that *Aedes*-transmitted LF can be eliminated, even with 5–6 rounds of MDA.

People with chronic disease condition have become very rare and no disease was found in younger generation. If any patient with chronic disease was found in any division of the country, the health centres and hospital will provide them quality services to alleviate the suffering in lymphedema and hydrocele patients. Thus, from the perspective of MMDP, Tonga met the LF elimination criterion.

Following the completion of all surveys and generation of proof that transmission was interrupted and chronic disease condition was very rare, Tonga submitted the dossier and successfully obtained WHO’s validation of elimination of LF as a public health problem in the year 2018.

The WHO guidelines suggest that, after validation of elimination of LF as a public health problem, surveillance activities should be continued to detect persistent LF infection foci, if any [13]. After the elimination of LF as a public health problem, the next milestone of the programme is the elimination of transmission, which envisages reduction of incidence of new LF infection to zero level. The MOH intends to hold discussions with stakeholders and regional forums to progress towards elimination of transmission of LF from the country.

## Data Availability

The national data presented in the manuscript belong to the Government of Tonga, but it can be accessed with the permission of the Ministry of Health.

## Abbreviations

Ag: Antigen
ALB: Albendazole
DEC: Diethylcarbamazine
EU: Evaluation unit
ICT: Immunochromatographic card test
IU: Implementation unit
LF: Lymphatic filariasis
M & E: Monitoring and evaluation
MDA: Mass drug administration
Mf: Microfilaria
MOH: Ministry of Health
PacELF: Pacific Programme to Eliminate Lymphatic Filariasis
WHO: World Health Organization

## References

[1] Sasa M (1976). Human Filariasis. A global survey of epidemiology and control.

[2] Desowitz RS, Berman SJ, Puloka T (1976). Hyperendemic subperiodic Bancroftian filariasis: a search for clinical and immunological correlates of microfilaraemia. Bull World Health Organ.

[3] Ottesen EA (2000) The global programme to eliminate lymphatic filariasis. Trop Med Int Hlth. 2000*;* 5: 591–594. 10.1046/j.1365-3156.2000.00620.x 11044272

[4] WHO/WPRO (2006). The PacELF way: towards elimination of lymphatic filariasis from the Pacific, 1999-2005.

[5] World Health Organization & Ministry of Health, Tonga (2012). Health Services Delivery Profile, Tonga.

[6] Buxton PA. Researches in Polynesia and Melanesia. Mem London Sch Hyg Trop Med 1927; Part 1-4 (Relating principally to Medical Entomology260 pp).

[7] Ramalingam A (1968). The epidemiology of filarial transmission in Samoa and Tonga. Ann Trop Med Parasit.

[8] Ramalingam A, Belkin NJ (1964). Vectors of sub-periodic bancroftian filariasis in the Samoa-Tonga area. Nature.

[9] Ramalingam A, Belkin NJ (1965). Mosquito studies III. The new *Aedes* from Tonga and Samoa. Contr Amer Entom Inst.

[10] Iyengar MOT (1965). Epidemiology of Filariasis in the South Pacific. South Pacific Comm Tech Paper No. 8 183 pp (mimeograph). South Pacific Commission, Noumea, New Caledonia. Aust J Entomol.

[11] WHO/SPC (1974). World Health Organization & South Pacific Commission. Report on the 4th Joint WHO/SPC Seminar on Filariasis and Vector Control, Apia, Western Samoa.

[12] Ministry of Health. Unpublished report. Ministry of Health. Government of Tonga. 1999.

[13] WHO (2011). Global programme to eliminate lymphatic filariasis. Monitoring and epidemiological assessment of mass drug administration. A manual for national elimination programmes.

[14] WHO (2017). Validation of elimination of lymphatic filariasis as a public health problem.

[15] Joseph H, Maiava F, Naseri T, Taleo F, Ake M, Capuano C, Melrose W. Application of the filariasis CELISA antifilarial IgG_4_ antibody assay in surveillance in lymphatic filariasis elimination programmes in the South Pacific. J Trop Med. 2011:492023. 10.1155/2011/492023 PMC3180782 21961018

[16] Chu BK, Gass K, Batcho W, Ake M, Dorkenoo AM, Adjinacou E, Mafi E, Addiss DG (2014). Pilot assessment of soil-transmitted helminthiasis in the context of transmission assessment surveys for lymphatic filariasis in Benin and Tonga. PLoS Negl Trop Dis.

[17] Esterre E, Plichart C, Sechan Y, Nguyen PL (2001). The impact of 34 years of massive DEC chemotherapy on Wuchereria bancrofti infection and transmission: the Maupiti cohort. Trop Med Int Hlth.

[18] Lau CL, Sheridan S, Ryan S, Roineau M, Andreosso A, Fuimaono S, Tufa J, Graves PM (2017). Detecting and confirming residual hotspots of lymphatic filariasis transmission in American Samoa 8 years after stopping mass drug administration. PLoS Negl Trop Dis.

[19] Southgate BA (1992). The siginificance of low density microfilaraemia in the transmission of lymphatic filarial parasites. J Trop Med Hyg.

[20] Ave C, Kapa DR, Ottesen EA (2018). Elimination of lymphatic filariasis as a public health problem from Cook Islands. Trop Med Health.

